# Identification of *Leishmania* spp. and *Trypanosoma cruzi* in bats captured in El Paso County, Texas

**DOI:** 10.1371/journal.pntd.0014169

**Published:** 2026-04-03

**Authors:** Juan C. Silva-Espinoza, Priscila S. G. Farani, Maria Fernanda Lopez, Edith Sandoval, Felipe Rodriguez, Kenneth A. Waldrup, Delfina C. Domínguez, Rosa A. Maldonado

**Affiliations:** 1 Department of Biological Sciences, The University of Texas at El Paso, El Paso, Texas, United States of America; 2 Department of Pharmaceutical Sciences, School of Pharmacy, The University of Texas at El Paso, El Paso, Texas, United States of America; 3 Texas Department of State Health Services, Zoonosis Control Region 10, El Paso, Texas, United States of America; 4 Department of Public Health Sciences, Clinical Laboratory Science, the University of Texas at El Paso, El Paso, Texas, United States of America; Advanced Centre for Chronic and Rare Diseases, INDIA

## Abstract

*Leishmania* spp*.* and *Trypanosoma cruzi* are protozoan parasites that cause leishmaniasis and Chagas disease, respectively. In the United States, autochthonous transmission cycles of both *Leishmania* and *T. cruzi* have been documented, particularly in the southern and southwestern regions. Previous studies in El Paso, Texas, have identified dogs, cats, and several sylvatic mammals as infected hosts for these pathogens; however, the role of bats has remained largely unexplored. Here, we conducted a cross-sectional, observational study of 29 wild bats, opportunistically collected from domestic and peridomestic urban environments in El Paso, Texas, as part of local rabies surveillance. DNA extracted from available heart, spleen, skin, and lung tissues was analyzed by quantitative PCR targeting *T. cruzi* satellite DNA and *Leishmania* 18S rDNA, followed by DNA sequencing for confirmation. PCR-based molecular detection identified *Leishmania* spp. in six bats (24.1%) and *Trypanosoma cruzi* in thirteen bats (44.8%). DNA sequencing analysis confirmed the presence of parasite-specific DNA in four *Leishmania*-positive samples and twelve *Trypanosoma cruzi*-positive samples. Restriction fragment length polymorphism (RFLP) analysis showed that the digestion patterns of the *T. cruzi* samples differed from those observed in the TcBat reference control. However, given the limited characterization and known genetic variability of the TcBat lineage, as well as the constraints of PCR-RFLP–based typing, these findings do not conclusively exclude the possibility that the detected strains belong to the TcBat genotype. Co-detection of both parasites was detected in 3 out of 18 *Tadarida brasiliensis* specimens. Mapping of capture sites showed infected bats occurring in both urban and suburban areas of El Paso County. This study provides evidence of the molecular detection of *Leishmania spp.* and *T. cruzi* in bats from El Paso, Texas. These findings highlight bats as sylvatic hosts for medically important trypanosomatids in the U.S.–Mexico border region and emphasize the need for expanded surveillance to assess zoonotic risk and its environmental drivers.

## Author summary

Leishmaniasis and Chagas disease affect millions of people in the Americas and other parts of the world. These diseases are mainly transmitted by insect vectors, the sand fly for *Leishmania,* and the kissing bug for *Trypanosoma cruzi*. Wild and domestic animals can also serve as reservoirs, maintaining the parasites in nature. In this study, we examined wild bats from El Paso County, Texas, a region along the United States–Mexico border that has been largely overlooked in terms of vector-borne disease surveillance. Using molecular techniques, we detected the presence of *Leishmania* and *T. cruzi* DNA in several bat species. Our findings suggest that bats in this area may play an important role in the natural transmission cycles of these parasites. This study highlights the need for continued monitoring of wildlife hosts and insect vectors in the border region to better understand the ecology and potential public health risks associated with these neglected tropical diseases.

## 1.  Introduction

*Leishmania* spp. and *Trypanosoma cruzi* are protozoan parasites of significant public health concern. They are recognized by the World Health Organization (WHO) as Neglected Tropical Diseases (NTDs) prioritized for global control and elimination efforts [1,2]. Both parasites have complex zoonotic cycles that involve wild and domestic mammalian hosts and insect vectors, facilitating their persistence and spread across diverse ecological settings in the Americas [3–5].

Bats (Chiroptera) have increasingly been recognized as important wildlife hosts and, in some contexts, potential sentinels for trypanosomatid parasites, including *T. cruzi* and *Leishmania* spp. [6–9]. Their ecological and behavioral traits (long lifespan, gregarious roosting, insectivorous or hematophagous diets, and wide migratory ranges) facilitate contact with vectors and other host species, making them important components of sylvatic transmission cycles [10]. Insectivorous bats like *Tadarida brasiliensis* (Mexican free-tailed bat) and *Lasiurus xanthinus* (western yellow bat), commonly found throughout the southwestern United States, often share habitats with triatomine bugs and sand flies, which may facilitate exposure to *T. cruzi* and *Leishmania* parasites [11–13]. Additionally, bats can serve as prey for carnivores such as domestic dogs, cats, and wild mesocarnivores, providing a plausible trophic route for oral transmission of *T. cruzi* [8].

In the last decade, research has increasingly focused on identifying *Leishmania* spp. and *T. cruzi* parasites in the U.S. Although autochthonous human cases of Chagas disease and leishmaniasis remain relatively uncommon in Texas, both parasites have been reported in the state for decades [14–19]. More than 60 locally acquired *T. cruzi* infections have been confirmed in humans, and serological surveys indicate a broader exposure risk among residents and animals [20]. Similarly, sporadic autochthonous cases of cutaneous leishmaniasis caused by *Leishmania mexicana* have been documented in southern and western Texas [18,21,22]. However, El Paso County remains largely underexplored regarding potential wildlife hosts, despite repeated detection of infected triatomine vectors in the area [2].

The U.S.–Mexico border region, including El Paso County, Texas, represents a unique ecological and epidemiological interface where desert, urban, and peridomestic habitats overlap. Previous studies have demonstrated *T. cruzi* infection in triatomine vectors, domestic animals, and wildlife within the region, suggesting active local transmission cycles [2,23]. In this study, a high proportion of triatomine vectors were infected with *T. cruzi*, and evidence of exposure was also detected in domestic dogs and wildlife, indicating widespread parasite circulation in the border area [2]. *Leishmania* DNA has also been detected in sand flies and dogs from adjacent areas, supporting the existence of sylvatic or peridomestic transmission foci [18,21]. Based on these dynamics, the present study aimed to investigate the presence of *Leishmania spp.* and *T. cruzi* in wild bats captured in El Paso County, Texas, and to explore their potential role as sylvatic hosts and possible reservoirs in local transmission cycles. Specifically, we sought to (i) molecularly detect kinetoplastid parasites in bat tissues, and (ii) determine the prevalence of *Leishmania* spp. and *T. cruzi* among different bat species. The present research provides new evidence on the potential involvement of insectivorous bat species in the epidemiology of trypanosomatid infections along the U.S.–Mexico border, an area of growing public health importance.

## 2.  Materials and methods

### 2.1 Ethics statement

The study protocol was approved by the Institutional Biosafety Committee (IBC) of the University of Texas at El Paso, protocol number 1659945–1, approved on 10/20/2020. IACUC protocol is not applicable, since the corpse bats were provided by the Texas Department of State Health Services (TDSHS), Zoonosis Control, and Animal Services, under the direction of Texas Parks and Wildlife permit number SPR/0316/066.

Wild bats were collected from domestic and peridomestic environments within urban areas of the El Paso region, Texas, from March to July 2021, during routine rabies surveillance conducted by a veterinarian from the Texas Department of State Health Services (TDSHS), Zoonosis Control, and Animal Services. This sampling was entirely opportunistic, i.e., the research team did not direct, select, request, or influence which bats were captured or euthanized. All collection and euthanasia were performed solely as part of TDSHS public health surveillance activities, independent of this study.

Bats were euthanized by carbon dioxide (CO₂) inhalation in accordance with federal and state public health guidelines and following the recommendations outlined in the 2020 American Veterinary Medical Association (AVMA) Guidelines for the Euthanasia of Animals. From these surveillance activities, only carcasses that tested negative for rabies were subsequently provided to the research team for molecular analysis.

Capture locations, including geographic coordinates and zip codes, as well as morphological characteristics of the specimens, were documented by TDSHS and shared with the investigators [24]. Sample collection and processing adhered to ethical guidelines and were conducted under protocol number 1659945–1, approved on October 20, 2020, by the Institutional Biosafety Committee (IBC). Additionally, bat collection was performed under the authority of Texas Parks and Wildlife permit number SPR/0316/066.

### 2.2 Tissue preparation and DNA extraction

A total of 29 bat carcasses, previously stored at -80 °C, were provided for analysis. Tissue samples from the heart, spleen, and/or skin were dissected from each bat and used for DNA extraction; however, availability varied among specimens. All three tissues were analyzed for both parasites when possible, details are provided in S1 Table. Organs were weighed and homogenized using the gentleMACS Dissociator (Miltenyi Biotec, Germany) in gentleMACS M-tubes with 200 µL of Tissue Lysis Buffer from High Pure PCR Template Preparation Kit (Roche) per 50 mg of tissue. Genomic DNA was then extracted from 50 mg of tissue using the High Pure PCR Template Preparation Kit (Roche, Cat. No. 11796828001) according to the manufacturer’s instructions. Briefly, lysed tissue homogenates were incubated with proteinase K and binding buffer for 10 minutes, and DNA was purified on silica-based filter columns through sequential washing and elution steps, yielding high-quality DNA suitable for downstream PCR analyses. Each extraction batch included a negative extraction control consisting of Guanidine-EDTA blood processed in parallel to monitor potential DNA contamination during the extraction procedure.

### 2.3 Detection of *Trypanosoma cruzi* and *Leishmania* spp. in bat tissues

Real-time PCR assays were performed using the TaqMan system (Applied Biosystems, Thermo Fisher Scientific, Waltham, MA, USA) to detect *T. cruzi* and *Leishmania* spp. DNA. For *T. cruzi*, the reaction targeted the satellite DNA sequence as previously described [25,26], using the primers Cruzi 1 (5′-ASTCGGCTGATCGTTTTCGA-3′) and Cruzi 2 (5′-AATTCCTCCAAGCAGCGGATA-3′) at a final concentration of 750 nM each, and the probe Cruzi 3 (5′-FAM-CACACTGGACACCAA-MGB-3′) at 250 nM using FastStart Universal Probe Master (Rox, Roche, Cat. No. 04914058001). This reaction amplified a 166 bp fragment specific to *T. cruzi*. Thermal cycling conditions assay consisted of an initial step at 95 °C for 10 min, followed by 40 cycles of denaturation at 95 °C for 15 sec and annealing/extension at 58 °C for 1 min. Appropriate positive controls (reference parasite DNA) and negative controls (nuclease-free water) were included in each run. For *Leishmania* detection, the 18S rDNA target was amplified following previous methodology [27], using primers 18S rDNA F (5′-GTACTGGGGCGTCAGAGGT-3′) and 18S rDNA R (5′-TGGGTGTCATCGTTTGCAG-3′), with the probe 18S rDNA Tq (5′-FAM-AATTCTTAGACCGCACCAAG-NFQ-MGB-3′) using FastStart Universal Probe Master (Rox, Roche, Cat. No. 04914058001). This assay amplifies a 155 bp fragment of the *Leishmania* 18S rDNA. As an endogenous control, TaqMan 20X Pre-made assay for 18S was used (Assay ID Hs99999901_s1), providing a 187 bp fragment. The thermal cycling conditions assay consisted of an initial step at 95 °C for 10 min, followed by 40 cycles of denaturation at 95 °C for 15 sec and annealing/extension at 60 °C for 1 min. Appropriate positive controls (reference parasite DNA) and negative controls (nuclease-free water) were included in each run, and each sample was run in duplicate. Amplifications were conducted on a QuantStudio 3 Real-Time PCR System (Applied Biosystems, Thermo Fisher Scientific, Waltham, MA, USA), and results were analyzed using the QuantStudio Design & Analysis Software. Threshold was set at 0.02 for both targets, and amplification for *T. cruzi* SatDNA or *Leishmania* 18S rDNA in either one of the replicates tested was considered positive. Amplification products were further verified by electrophoresis using E-Gel 2% agarose gels with 48 wells (Thermo Fisher Scientific, Waltham, MA, USA). Samples were mixed in a 1:1 ratio with the supplied loading buffer before loading, and gels were run according to the manufacturer’s protocol. Gel images were captured using the Thermo Fisher gel documentation system.

PCR products yielding positive amplicons were excised from agarose gels and purified using the Wizard SV Gel and PCR Clean-Up System (Promega, Madison, WI, USA), following the manufacturer’s instructions. Purified amplicons were then submitted for sequencing using the Plasmidsaurus PCR Premium Standard service (Plasmidsaurus, Eugene, OR, USA), which uses long-read Oxford Nanopore sequencing. Library preparation was performed by the provider and included end-repair and barcode ligation, pooling, and sequencing on a MinION flow cell. Because molecular detection in this study was determined by validated qPCR detection with gel-confirmed amplicons, sequencing was used as an orthogonal confirmation of amplicon identity rather than as the sole criterion for detection. Resulting sequence data were queried against the NCBI BLAST nucleotide database and TriTrypDB for species identification, and identity was confirmed based on alignment coverage and percent identity [2].

### 2.4 *Trypanosoma cruzi* TcBat genotyping analysis

Samples testing positive for *T. cruzi* were further analyzed for the DTU TcBat genotype as previously described [28]. PCR amplification of an 832 bp fragment of the TcSC5D gene was performed using primers TcSC5D-fwd (5’-GGACGTGGCGTTTGATTTAT-3’) and TcSC5D-rev (5’-TCCCATCTTCTTCGTTGACT-3’). Each reaction contained 200 ng of DNA, 10 pmol of each primer, and 1 × GoTaq Green Master Mix (Promega Corporation, Madison, WI, USA). PCR conditions were as follows: initial denaturation at 94°C for 4.5 min, followed by 35 cycles of denaturation at 94°C for 30 sec, annealing at 58°C for 30 sec, and extension at 72°C for 30 sec, with a final extension at 72°C for 7 min. Restriction fragment length polymorphism (RFLP) analysis was performed by digesting the PCR products with 1 U of *FastDigest PaeI* and 1 U of *FastDigest KspAI* (Thermo Fisher Scientific, Waltham, MA, US) for 1 hour at 37°C. Digested products were analyzed by electrophoresis on a 1.5% agarose gel.

### 2.5 Geographic mapping of bat collection sites

The locations of wild bats that tested positive for *Leishmania spp.* and/or *T. cruzi* were documented. The TDSHS provided capture coordinates and zip codes. Geographic mapping of the collection sites was performed using ArcGIS Pro (Esri, USA) with a public-domain basemap from the U.S. Geological Survey (USGS) (https://www.usgs.gov). Positive sampling points were overlaid onto this basemap to visualize the spatial distribution of infected bats across El Paso County, Texas.

## 3.  Results

### 3.1. Prevalence of *Leishmania* spp. and *Trypanosoma cruzi* in collected bats

This study was a cross-sectional, observational investigation conducted over six months using opportunistically collected wild bat specimens in El Paso, Texas. These bats were collected from domestic and peridomestic environments within urban areas of the county’s urban areas. Tissue samples were obtained for molecular analysis, including the heart, spleen, and skin. Skin tissue was analyzed only in bats presenting visible dermal lesions or abnormalities at necropsy. Therefore, detection of dermotropic typanosomatids in skin samples reflects lesion-associated sampling rather than systematic screening of all individuals. As a result, the presence and frequency of trypanosomatids in skin tissue may be biased toward symptomatic bats, and parasite absence in skin cannot be interpreted as evidence of a lack of dermal infection in an asymptomatic individual. These tissues were selected based on their known relevance to the pathology of Leishmania spp. and T. cruzi manifestations. A total of 29 bats were collected from El Paso, Texas, representing six species from the families Molossidae and Vespertilionidae (S1 Table). The most frequently captured species was Tadarida brasiliensis (Mexican free-tailed bat), which accounted for 18/29 (62.1%) of all collected bats, followed by Myotis velifer (3/29, 10.3%), Parastrellus hesperus (3/29, 10.3%), Lasiurus xanthinus (2/29, 6.9%), Lasionycteris noctivangans (2/29, 6.9%), and Eptesicus fuscus (1/29, 3.4%).

PCR screening revealed that 6/29 bats (24.1%) tested positive for Leishmania spp., while 13/29 (44.8%) were positive for T. cruzi (Table 1). Among T. brasiliensis, 3/18 (16.6%) tested positive for Leishmania spp. and 7/18 (38.9%) for T. cruzi.

**Table 1:** The proportion of Leishmania spp. and T. cruzi in wild bats. Species represented by ≤ 3 individuals are included for descriptive purposes only.

Bat Family	Bat Species	# Tested	*Leishmania spp.*		*T. cruzi*	
			# Positive	Proportion	# Positive	Proportion
Molossidae	*Tadarida brasiliensis*	18	5	27.8%	10	72.2%
Vespertilionidae	*Eptesicus fuscus*	0	0	0.0%	0	0.0%
	*Lasionycteris noctivagans*	2	0	0.0%	1	50.0%
	*Lasiurus xanthinus*	2	0	0.0%	1	50.0%
	*Myotis velifer*	3	1	33.3%	0	0.0%
	*Parastrellus hesperus*	3	0	0.0%	1	50.0%
	Total	29	6	24.1%	13	44.8%

### 3.2 DNA sequencing analysis

For *Leishmania* spp*.*, 6 samples tested positive by qPCR-positive samples, of which 4 yielded high-quality sequencing (Table 2, S2 Table)*.* Species identification was attempted using the protocol described by Schönian et al. (2013) using RFLP methodology [29]. However, the *Leishmania* species could not be confidently determined based on the obtained band patterns due to low-quality band patterns and a lack of representative positive controls. Therefore, the positive samples are being conservatively reported here as *Leishmania* spp. In contrast, 13 bats tested positive for *T. cruzi* by qPCR, and sequencing successfully confirmed parasite identity in 12 of these samples (Table 2, S2 Table).

Additionally, all *T. cruzi*-positive samples were subjected to amplification of the TcSC5D target followed by restriction enzyme treatment to determine the presence of the TcBat DTU, commonly associated with bats. However, none of the sequenced samples exhibited the expected TcBat digestion pattern. The observed RFLP profiles were insufficient for reliable DTU classification, precluding conclusions regarding similarity to TcI or other DTUs reported in the region [2,30].

**Table 2:** Molecular identification of *Leishmania spp.* and *Trypanosoma cruzi* in wild bats from El Paso, Texas, based on sequence alignment (E-value and percent identity). Sequences obtained from PCR amplicons were aligned with GenBank reference sequences to determine organism identity. E-values and percent identity values from BLASTn analyses are shown for each bat sample. Bat species are listed by family.

Bat Family	Bat ID	Bat Species	Organism	E-Value	Identity
Molossidae	R20-064	*Tadarida brasiliensis*	*Trypanosoma cruzi*	1.00E-81	100.00%
	R20-154	*Tadarida brasiliensis*	*Trypanosoma cruzi*	1.00E-76	98.17%
	R20-158	*Tadarida brasiliensis*	*Trypanosoma cruzi*	7.00E-80	99.39%
	R20-180	*Tadarida brasiliensis*	*Trypanosoma cruzi*	3.00E-78	98.78%
	R21-014	*Tadarida brasiliensis*	*Trypanosoma cruzi*	7.00E-80	99.39%
	R21-020	*Tadarida brasiliensis*	*Trypanosoma cruzi*	1.00E-81	100.00%
	R21-026	*Tadarida brasiliensis*	*Trypanosoma cruzi*	7.00E-80	99.39%
	R21-042*	*Tadarida brasiliensis*	*Trypanosoma cruzi*	7.00E-75	97.56%
	R21-068*	*Tadarida brasiliensis*	*Trypanosoma cruzi*	1.00E-76	98.17%
	R21-078*	*Tadarida brasiliensis*	*Trypanosoma cruzi*	7.00E-80	99.39%
	R21-023	*Tadarida brasiliensis*	*Leishmania* spp.	1.00E-81	100.00%
	R21-068*	*Tadarida brasiliensis*	*Leishmania* spp.	1.00E-76	98.17%
	R21-078*	*Tadarida brasiliensis*	*Leishmania* spp.	7.00E-80	99.39%
	R21-014	*Tadarida brasiliensis*	*Leishmania* spp.	N/A	N/A
	R21-042*	*Tadarida brasiliensis*	*Leishmania* spp.	N/A	N/A
Vespertilionidae	R21-021	*Lasiurus xanthinus*	*Trypanosoma cruzi*	2.00E-80	99.39%
	R21-043	*Lasionycteris noctivangans*	*Trypanosoma cruzi*	N/A	N/A
	R20-062	*Parastrellus hesperus*	*Trypanosoma cruzi*	7.00E-80	99.39%
	R20-066	*Myotis velifer*	*Leishmania spp.*	8.00E-44	87.82%

*Specimen with co-detection of Leishmania spp. and T. cruzi.

### 3.3 Geographic distribution of infected bats

**Fig 1:**
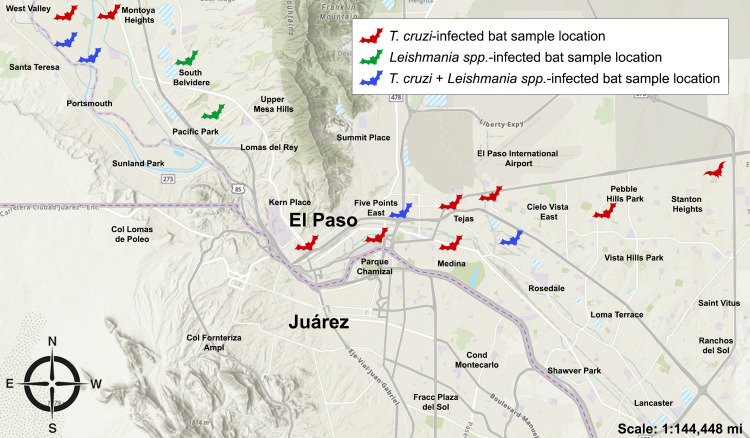
Geographic distribution of molecular detection of Leishmania spp. and Trypanosoma cruzi in bats in the El Paso region. The map shows the locations of bats that tested positive for Leishmania spp. and/or T. cruzi in El Paso, Texas, and surrounding areas. Each icon represents a bat sample collected and tested in this study. Red icons indicate bats that tested positive for T. cruzi. Green icons indicate bats that tested positive for Leishmania spp. Blue icons indicate bats that tested positive for both T. cruzi and Leishmania spp. Bats were detected across multiple locations, including urban, suburban, and sylvatic environments, with potential implications for zoonotic transmission. Base map source: **U.**S. Geological Survey (public domain; https://www.usgs.gov). Bat icons: obtained from Openclipart (public domain, CC0 license).

The locations of bats that tested positive for Leishmania spp. and/or T. cruzi were mapped based on the collection site coordinates (Fig 1). PCR-positive bats were detected at collection sites spanning from the northwest to the southeast of El Paso, including highly populated areas that encompass a mix of urban, suburban, and adjacent sylvatic settings [31]. Several positive bats were collected near human-populated areas; however, due to opportunistic sampling and the absence of mapped negative samples, spatial patterns cannot be reliably inferred. Further surveillance studies are needed to determine whether these bats may be involved in local transmission cycles.

## 4. Discussion

In this cross-sectional study of 29 collected bats from domestic and peridomestic environments in El Paso County, we provide the first molecular evidence of *Leishmania* spp. and *Trypanosoma cruzi* infection in local bat populations. Overall, 6/29 bats (24.1%) were positive for *Leishmania* spp. and 13/29 (44.8%) were positive for *T. cruzi* by qPCR, with sequencing confirmation obtained for 4 out of 5 *Leishmania*-positive samples and 12 out of 13 of *T. cruzi*-positive samples. These bats represented six species across two families (Molossidae and Vespertilionidae), and the collection was dominated by *Tadarida brasiliensis* (18/29; 62.1%). Within *T. brasiliensis*, molecular detection was common, with 5/18 (27.8%) positive for *Leishmania* spp. and 10/18 (72.2%) positive for *T. cruzi*, and co-infections were also observed in specimens. These findings extend prior evidence of intense local transmission cycles in the region and support the inclusion of bats as potential sylvatic hosts contributing to the ecology of trypanosomatid infections along the U.S.–Mexico border. About 14 different bat species have been reported in El Paso, TX. In 2000, *Antrozous pallidus* (Pallid bat), *Pipistrellus hesperus* (Western Pipistrelle), and the *Tadarida brasiliensis* (Mexican free-tailed bat) were identified as the most common bat species present in the Greater El Paso region [32,33]. In our sampling, after *T. brasiliensis*, the next most frequent species were *Myotis velifer* (3/29; 10.3%) and *Parastrellus hesperus* (3/29; 10.3%), followed by *Lasiurus xanthinus* and *Lasionycteris noctivagans* (each 2/29; 6.9%), and *Eptesicus fuscus* (1/29; 3.4%). During migration, *Lasiurus xanthinus* heads to Mexico, while *Tadarida brasiliensis* migrates to Mexico and Central America [34,35]. As the effects of climate change intensify, the migration period for these bats may extend, potentially influencing disease transmission patterns. Moreover, changes in temperature or precipitation affect some insectivore species, especially in maternity roosts, where water and warm conditions are essential for females during pregnancy and lactation [36]. Because these species are insectivorous, their feeding ecology plausibly increases contact with infected arthropods (e.g., triatomines for *T. cruzi* and sand flies for *Leishmania*), supporting biologically plausible routes of exposure and maintenance even when animals are sampled in urban/peridomestic settings [7,9,10,37,38].

This is the first study in El Paso showing bats with molecular detection of *Leishmania* spp*.* and/or *T. cruzi*. Bats are known to share habitats with sandfly vectors, particularly in caves, crevices, and tree roots. While vector bites may occur, is more plausible that insectivorous bats may become infected with *Leishmania* spp*.* through the oral ingestion of infected sandflies, a route that has been experimentally demonstrated and proposed as a natural mode of transmission in wildlife reservoirs [39–41]. Experimental and field studies have shown that bats are competent hosts that can harbor *Leishmania* parasites, with evidence of both parasite DNA and seroconversion in wild populations [42,43]. Given their extensive foraging range and seasonal migration, bats may encounter endemic foci of *Leishmania* during movement across regions, potentially serving as sylvatic hosts [44,45].

Phylogenetic analysis could, in principle, provide insight into the genetic relationships of the *Leishmania* parasites detected in bats in this study. However, the amplicons obtained were relatively short and of insufficient quality to support robust phylogenetic reconstruction. The inability to obtain readable sequences from 2 *Leishmania*-positive and 1 *T. cruzi*-positive samples is likely attributable to low parasite DNA concentrations, as reflected by high Ct values for those samples (R21-014: 36.15 ± 0.66; R21-042: 33.64 ± 0.14; R21-043: 36.20 ± 0.96), which commonly limit downstream sequencing performance in field-collected specimens with low parasitemia. Similar limitations have been reported in previous field studies, where low parasite loads and partial amplification hindered sequence alignment and comparative analyses [42,46]. For this reason, the results were conservatively reported as *Leishmania* spp., rather than assigning uncertain species-level identities. This limitation is acknowledged in the present study, and future investigations using multilocus sequence typing (MLST) or whole-genome sequencing will be necessary to more accurately characterize *Leishmania* detection in bat populations [29].

Previous ecological and molecular studies have demonstrated that insectivorous bat species frequently encounter and ingest triatomines, reinforcing their potential role in parasite maintenance cycles [47]. In 2021, Bergner *et al.* demonstrated that *T. cruzi* parasites could also be found in the saliva of neotropical bats, which could associate bats as both reservoirs and transmitters of *T. cruzi* infection [8]. Bats’ predators include barn owls, domestic dogs**,** and domestic/feral cats [38,48,49]. Our detection of *T. cruzi* in bats from El Paso is consistent with the high infection pressure documented in the local transmission cycle. Previous studies from our laboratory reported *T. cruzi* infection in 67.4% of triatomine bugs and seropositivity in 8.4% of domestic dogs and 7.3% of wild mammals in the El Paso–southern New Mexico region [2]. More recent surveillance indicates a substantial increase in vector infection, with 89% of triatomines testing positive in the same region, representing one of the highest prevalence rates reported in the United States (accepted for publication). This intense transmission cycle contrasts with results from large-scale surveys elsewhere in the country, which reported <1% prevalence in bats [12] or a single infected *Tadarida brasiliensis* out of 283 examined [13]. The discrepancy may reflect a combination of ecological and methodological factors that include elevated host infection pressure in the border region, greater oral transmission risk in specific areas where vectors are abundant, and the use of a high-sensitivity qPCR assay targeting satellite DNA, in contrast to single-copy gene PCRs used in earlier studies. Collectively, these factors likely account for the higher *T. cruzi* prevalence observed in our study [11–13]. However, we acknowledge that the variable availability of tissues among individual bats represents a limitation of this study, as differences in the number and type of tissues analyzed per animal may have influenced detection probability and, consequently, prevalence estimates.

The detection of *T. cruzi* in bat samples without the band pattern characteristic of the TcBat genotype suggests several possibilities. TcBat is a DTU of *T. cruzi* first described in bats and is considered bat-associated due to its frequent detection in chiropteran hosts across Latin America [50,51]. However, it is well established that bats can also harbor other DTUs, particularly TcI, which is highly prevalent across sylvatic, peridomestic, and domestic transmission cycles throughout the Americas [10,52]. Cross-species transmission is another plausible explanation. While this study did not assess transmission pathways or interactions with other animal hosts, previous experimental studies have shown that trophic transmission through ingestion of infected triatomine vectors or their feces can occur in mammalian hosts [47]. Additionally, geographical variation in DTU prevalence may contribute to the observed findings. Although TcBat has been identified in regions of South and Central America, its prevalence appears lower or absent in some northern regions, including parts of the southern United States and northern Mexico, where TcI predominates [53]. A final consideration is the potential for genetic variation within the TcBat lineage itself. Mutations or recombination events could alter the restriction enzyme recognition sites used in RFLP assays, potentially leading to false negatives or misclassification of TcBat isolates. This possibility underscores the limitations of PCR-RFLP typing alone and highlights the need for sequencing-based approaches, such as multilocus sequence typing (MLST) or whole-genome analysis, to accurately resolve DTU identity and intra-lineage diversity. Interestingly, phylogenetic analyses have shown that TcBat shares a more recent common ancestor with TcI than with other DTUs (TcII–TcVI), suggesting a closer evolutionary relationship between these two lineages [54]. This phylogenetic proximity may be reflected in shared biological characteristics, such as host tropism, transmission dynamics, and tissue distribution. Collectively, these findings emphasize the need for further molecular epidemiological studies to better understand the diversity and transmission ecology of *T. cruzi* lineages in bat populations of the southwestern United States.

Recent increases in *T. cruzi* surveillance in humans and animals in the United States reflect growing recognition of Chagas disease as an emerging public health concern, particularly in the southeastern and southwestern regions, including Texas and Arizona [53,55]. Once considered a tropical or travel-associated disease primarily, Chagas disease is now increasingly recognized as an autochthonous infection in the southern United States, where triatomine vectors and mammalian hosts are well established in domestic and peridomestic settings [2,15,16,56]. Within this context, the current findings indicate that chiropteran species potentially play a role in the sylvatic transmission cycles of *Leishmania* spp. and *T. cruzi* within the El Paso region, an area characterized by ecological and geographical variability along the U.S.–Mexico border. The mobility of wildlife and favorable environmental conditions may facilitate parasitic persistence and cross-species transmission, highlighting the critical need to elucidate the role of wildlife hosts in local disease ecology.

In a broader One Health and epidemiological context, higher-resolution molecular typing would strengthen interpretation of both trypanosomatid detections reported here by clarifying (i) whether the *Leishmania* sequences identified in the El Paso region are most consistent with endemic North American lineages or instead reflect introduced lineages more often associated with Old World foci in Africa, the Middle East, or South Asia [57], and (ii) whether *T. cruzi* molecular detection in local bats reflect circulation of established regional DTUs versus repeated introductions linked to animal movement and cross-border ecology. Such analyses would improve inference regarding geographic origins, transmission pathways, and public health relevance. Given El Paso’s high connectivity, particularly the presence of Fort Bliss, a major U.S. military installation and international training hub, the ongoing movement of personnel, working animals, and equipment between endemic regions and U.S. training sites remains a plausible mechanism for inadvertent translocation of *Leishmania* parasites and/or sand fly vectors, as previously proposed [44,45]. Collectively, these findings support the need for integrated molecular surveillance that links wildlife, vectors, domestic animals, and human exposure data to better define local transmission dynamics and to assess the risk of introduction or establishment of non-endemic trypanosomatid lineages in the U.S.–Mexico border region. From a public health perspective, they also underscore the importance of enhanced awareness campaigns in border communities focused on vector avoidance and zoonotic disease prevention, including practical guidance on pest control, proper pet hygiene, and routine veterinary care for animals with potential exposure to wildlife hosts.

## Supporting information

S1 Fig Agarose gel electrophoresis of PCR amplification products for *T. cruzi* satellite DNA, *Leishmania* 18S rDNA, and mammalian 18S rRNA gene. PCR products were resolved on a 2% agarose gel pre-stained with SYBR Safe and visualized under UV illumination. The expected amplification fragment sizes were 166 bp for *T. cruzi* satellite DNA, 155 bp for *Leishmania* 18S rDNA, and 187 bp for mammalian 18S rRNA. Lanes 1–10 represent representative bat tissue samples; “C-” indicates negative extraction controls.
(TIF)

S1 Table Bat species, collection sites, sampled tissues, and molecular detection of *Leishmania* spp. and *Trypanosoma cruzi* in wild bats captured in El Paso County, Texas. (DOCX)

S2 Table DNA sequencing identification of *Leishmania* spp. and *Trypanosoma cruzi* in bat tissue samples. (DOCX)
