# Correction: Identification of *Leishmania* spp. and *Trypanosoma cruzi* in bats captured in El Paso County, Texas

**DOI:** 10.1371/journal.pntd.0014417

**Published:** 2026-06-09

**Authors:** Juan C. Silva-Espinoza, Priscila S. G. Farani, Edith Sandoval, Maria Fernanda Lopez, Felipe Rodriguez, Kenneth A. Waldrup, Delfina C. Domínguez, Rosa A. Maldonado

The authors are listed out of order. Please view the correct author order, affiliations, and citation here:

Juan C. Silva-Espinoza^1^, Priscila S. G. Farani^1,2^, Edith Sandoval^1^, Maria Fernanda Lopez^1^, Felipe Rodriguez^1^, Kenneth A. Waldrup^3^, Delfina C. Domínguez^4^, Rosa A. Maldonado^1^

**1** Department of Biological Sciences, The University of Texas at El Paso, El Paso, Texas, United States of America, **2** Department of Pharmaceutical Sciences, School of Pharmacy, The University of Texas at El Paso, El Paso, Texas, United States of America, **3** Texas Department of State Health Services, Zoonosis Control Region 10, El Paso, Texas, United States of America, **4** Department of Public Health Sciences, Clinical Laboratory Science, the University of Texas at El Paso, El Paso, Texas, United States of America

Silva-Espinoza JC, Farani PSG, Sandoval E, Lopez MF, Rodriguez F, Waldrup KA, et al. (2026) Identification of Leishmania spp. and Trypanosoma cruzi in bats captured in El Paso County, Texas. PLoS Negl Trop Dis 20(4): e0014169. https://doi.org/10.1371/journal.pntd.0014169
