# Supplementary figures and images for: Identification of Leishmania spp. and Trypanosoma cruzi in bats captured in El Paso County, Texas
Source: PLoS Negl Trop Dis. 2026 Apr 3;20(4):e0014169. doi: 10.1371/journal.pntd.0014169 (PMC13061320; doi:10.1371/journal.pntd.0014169)

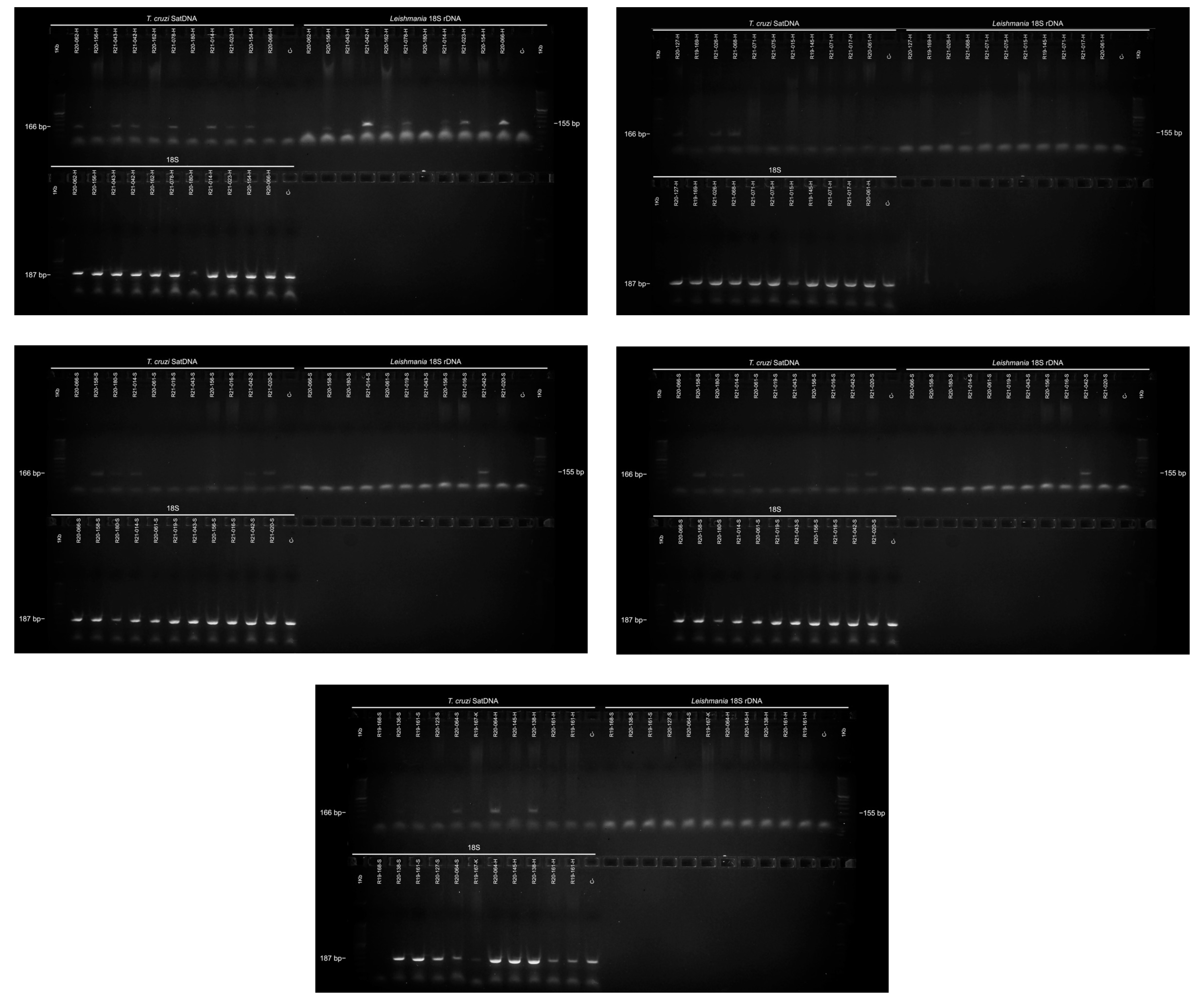

Supplement: S1 Fig — PCR products were resolved on a 2% agarose gel pre-stained with SYBR Safe and visualized under UV illumination. The expected amplification fragment sizes were 166 bp for T. cruzi satellite DNA, 155 bp for Leishmania 18S rDNA, and 187 bp for mammalian 18S rRNA. Lanes 1–10 represent representative bat tissue samples; “C-” indicates negative extraction controls. (TIF) [file pntd.0014169.s001.tif]
