# Supplementary material for: Identification of Leishmania spp. and Trypanosoma cruzi in bats captured in El Paso County, Texas
Source: PLoS Negl Trop Dis. 2026 Apr 3;20(4):e0014169. doi: 10.1371/journal.pntd.0014169 (PMC13061320; doi:10.1371/journal.pntd.0014169)
Supplement: S1 Table — (DOCX) [file pntd.0014169.s002.docx]

**Supplementary Table 1:** Bat species, collection sites, sampled tissues, and molecular detection of *Leishmania spp. and Trypanosoma cruzi* in wild bats captured in El Paso County, Texas.

| **Bat ID** | **Bat species** | **GPS Coordinates^a^** | **Heart^b^** | **Spleen^b^** | **Skin^b^** | **Lungs^b^** | ***T. cruzi*^b^** | ***Leishmania spp.*^b^** |
| --- | --- | --- | --- | --- | --- | --- | --- | --- |
| R20-162 | *Eptesicus fuscus* | 31.551259269134327, -106.1505496621775 | x | x |  |  |  |  |
| R19-169 | *Lasionycteris noctivangans* | 31.956433283199733, -106.40666335395123 | x | x |  |  |  |  |
| R21-043 | *Lasionycteris noctivangans* | 31.772538001085934, -106.38353781733353 | x | x |  |  | x |  |
| R21-019 | *Lasiurus xanthinus* | 31.846555367188344, -106.54879016269372 |  | x |  |  |  |  |
| R21-021 | *Lasiurus xanthinus* | 31.766319364513432, -106.44450387315345 |  | x |  |  | x |  |
| R19-145 | *Myotis velifer* | 31.693096638894193, -106.33789681918906 | x |  |  |  |  |  |
| R20-066 | *Myotis velifer* | 31.8378698397947, -106.52310719198087 | x | x | x | x |  | x |
| R21-016 | *Myotis velifer* | 31.76719122236596, -106.32286377685848 |  | x |  |  |  |  |
| R20-062 | *Parastrellus hesperus* | 31.796089530413916, -106.27039290569348 | x | x |  | x | x |  |
| R20-136 | *Parastrellus hesperus* | 31.786660489582022, -106.20138277184358 |  | x |  |  |  |  |
| R21-071 | *Parastrellus hesperus* | 31.79847576299467, -106.26582908849663 | x |  |  |  |  |  |
| R19-167 | *Tadarida brasiliensis* | 31.872185094147287, -106.62943062696685 |  |  | x |  |  |  |
| R20-061 | *Tadarida brasiliensis* | 31.827047963191045, -106.24178033397398 |  | x |  |  |  |  |
| R20-064 | *Tadarida brasiliensis* | 31.766840198042434, -106.48888193995565 | x | x |  |  | x |  |
| R20-123 | *Tadarida brasiliensis* | 31.79276863664042, -106.45465414616892 |  | x |  |  |  |  |
| R20-127 | *Tadarida brasiliensis* | 31.79649260477589, -106.16328844457828 | x | x |  |  |  |  |
| R20-154 | *Tadarida brasiliensis* | 31.790298657287924, -106.38520196151329 | x |  |  |  | x |  |
| R20-156 | *Tadarida brasiliensis* | 31.79599460981049, -106.38521290677753 | x | x |  |  |  |  |
| R20-158 | *Tadarida brasiliensis* | 31.782466847289985, -106.4188412955596 |  | x |  |  | x |  |
| R20-180 | *Tadarida brasiliensis* | 31.862144569082712, -106.61616676870617 | x | x |  |  | x |  |
| R21-014 | *Tadarida brasiliensis* | 31.827302128656566, -106.59240854744473 | x | x | x |  | x | x |
| R21-017 | *Tadarida brasiliensis* | 31.735992366447597, -106.33004580199052 | x | x |  | x |  |  |
| R21-020 | *Tadarida brasiliensis* | 31.867293220619672, -106.58083906676778 |  | x |  |  | x |  |
| R21-023 | *Tadarida brasiliensis* | 31.775768927723746, -106.44321763621053 | x | x |  |  | x | x |
| R21-026 | *Tadarida brasiliensis* | 31.781919416660262, -106.33449268849714 | x | x | x |  | x |  |
| R21-042 | *Tadarida brasiliensis* | 31.772538001085934, -106.38353781733353 | x | x |  |  | x | x |
| R21-068 | *Tadarida brasiliensis* | 31.83183744811796, -106.54257083452825 | x | x |  |  |  | x |
| R21-075 | *Tadarida brasiliensis* | 31.89064003405075, -106.57871447500145 | x | x |  | x |  |  |
| R21-078 | *Tadarida brasiliensis* | 31.862494030196153, -106.59581414616633 | x | x |  |  | x | x |

**a** Geographic coordinates correspond to capture locations provided by the Texas Department of State Health Services (TDSHS).

**b** “x” denotes tissues collected and/or samples testing positive by PCR for the corresponding parasite.
