# Supplementary material for: Identification of Leishmania spp. and Trypanosoma cruzi in bats captured in El Paso County, Texas
Source: PLoS Negl Trop Dis. 2026 Apr 3;20(4):e0014169. doi: 10.1371/journal.pntd.0014169 (PMC13061320; doi:10.1371/journal.pntd.0014169)
Supplement: S2 Table — (DOCX) [file pntd.0014169.s003.docx]

**Supplementary Table 2:** DNA sequencing identification of *Leishmania spp.* and *Trypanosoma cruzi* in bat tissue samples.

| **Bat ID** | **Bat Species** | **Sequence** | **Organism** | **E-Value** | **Identity %** |
| --- | --- | --- | --- | --- | --- |
| R21-043 | *Lasionycteris noctivangans* | - | *Trypanosoma cruzi* | N/A | N/A |
| R21-021 | *Lasiurus xanthinus* | TAATTCCTCCAAGCAGCGGATAGTTCAGGGTTGTTTGGTGTCCAGTGTGTGAACACGCAAACAGATATTGACAGAGAGTGCCTCTGACTCCCACCATTCATAATCGGAAACAAAAATTTGGACCACAACGTGTGATGCAGCAGCCGCTCGAAAACGATCAGCCGATT | *Trypanosoma cruzi* | 2.00E-80 | 99.39% |
| R20-062 | *Parastrellus hesperus* | AATCGGCTGATCGTTTTCGAGCGGCCGCTGCATCACACGTTGTGGTCCAAATTTTTGTTTCCGATTATGAATGGTGGGAGTCAGAGACACTCTCTGTCAATATCTGTTTGCGTGTTCACACACTGGACACCAAACAACCCTGAACTATCCGCTGCTTGGAGGAATTA | *Trypanosoma cruzi* | 7.00E-80 | 99.39% |
| R20-064 | *Tadarida brasiliensis* | AATTCCTCCAAGCAGCGGATAGTTCAGGGTTGTTTGGTGTCCAGTGTGTGAACACGCAAACAGATATTGACAGAGAGTGCCTCTGACTCCCACCATTCACAATCGGAAACAAAAATTTGGACCACAACGTGTGATGCAGCAGCCGCTCGAAAACGATCAGCCGATT | *Trypanosoma cruzi* | 1.00E-81 | 100.00% |
| R20-154 | *Tadarida brasiliensis* | TAATTCCTCCAAGCAGCGGATAGTTCAGGGTTGTTTGGTGTCCAGTGTGTGCACACGCAAACATACATTGAAAGAGACTGTCTCTGACTCCCGCCATTCATAATTCAAAACAAAAACATAGACCACAACGTGTGGTGCAGCGGCCGCTCGAAAACGATCAGCCGATT | *Trypanosoma cruzi* | 1.00E-76 | 98.17% |
| R20-158 | *Tadarida brasiliensis* | AATCGGCTGATCGTTTTCGAGCGGCTGCTGCATCACACGTTGTGGTCTAAATTTTTGTTTCCGATTGTGAATGGTGGGAGTCAGAGACACTCTCTGTCAATATCTGTTTGCGTGTTCACACACTGGACACCAAACAACCCTGAACTATCCGCTGCTTGGAGGAATTA | *Trypanosoma cruzi* | 7.00E-80 | 99.39% |
| R20-180 | *Tadarida brasiliensis* | TAATTCCTCCAAGCAGCGGATAATTCAGGGTTGTTTGGTGTCCAGTGTGTGCACACGCAAACATACATTGAAAGAGAGTGTCTCTGACTCCCGCCATTCATAATTCAAAACAAAAATTTAGACCACAGCGTGTGGTGCAGCAGCCGCTCGAAAACGATCAGCCGATT | *Trypanosoma cruzi* | 3.00E-78 | 98.78% |
| R21-014 | *Tadarida brasiliensis* | AATTCCTCCAAGCAGCGGATAATTCAGGGTTGTTTGGTGTCCAGTGTGTGAACACGCAAACAGATATTGACAGAGAGTGCCTCTGACTCCCACCATACACAATCGGAAACAAAAATTTGGACCACAACGTGTGATGCAGCGGCCGCTCGAAAACGATCAGCCGATTA | *Trypanosoma cruzi* | 7.00E-80 | 99.39% |
| R21-020 | *Tadarida brasiliensis* | TAATTCCTCCAAGCAGCGGATAGTTCAGGGTTGTTTGGTGTCCAGTGTGTGAACACGCAAACAGATATTGACAGAGAGTGTCTCTGACTCCCACCATTCATAATCGGAAACAAAAATTTGGACCACAACGTGTGATGCAGCAGCCGCTCGAAAACGATCAGCCGATT | *Trypanosoma cruzi* | 1.00E-81 | 100.00% |
| R21-026 | *Tadarida brasiliensis* | AATTCCTCCAAGCAGCGGATAATTCAGGGTTGTTTGGTGTCCAGTGTGTGAACACGCAAACAGATAGTGACAGAGAGTGTCTCTGACTCCCACCATTCACAATCGGAAACAAAAATTTGGACCACAACGTGTGATGCAGCAGCCGCTCGAAAACGATCAGCCGATT | *Trypanosoma cruzi* | 7.00E-80 | 99.39% |
| R21-042* | *Tadarida brasiliensis* | TAATTCCTCCAAGCAGCGGATAGTTCAGGGTTGTTTGGTGTCCAGTGTGTGAACACGCAAACAGATATTGACAGAGTGTGTCTCTGACTCCCACCATTCATAATTGGAAACAAAAATTTAGACCACAACGTGTGATGCAACAGCCGCTCGAAAACGATCAGCCGATT | *Trypanosoma cruzi* | 7.00E-75 | 97.56% |
| R21-068* | *Tadarida brasiliensis* | AATTCCTCCAAGCAGCGGATAATTCAGGGTTTTTTGGTGTCCAGTGTGTGAACACGCAAACAGATATTGAAAGAGAGTGCCTCTGACTCCCACCATACACAATCGGAAACAAAAATTTGGACCACAACGTGTGATGCAGCGGCCGCTCGAAAACGATCAGCCGATTA | *Trypanosoma cruzi* | 1.00E-76 | 98.17% |
| R21-078* | *Tadarida brasiliensis* | AATCGGCTGATCGTTTTCGAGCGGCTGCTGCATCACACGTTGTGGTCCAAATTTTTGTTTCCGATTGTGAATGGTGGGAGTCAGAGGACACTCTCTGTCAATATCTGTTTGCGTGTTCACACACTGGACACCAAACAACCCTGAACTATCCGCTGCTTGGAGGAATT | *Trypanosoma cruzi* | 7.00E-80 | 99.39% |
| R20-066 | *Myotis velifer* | GTACTGGGGCGTCAGAGGTGAAATTTTTAGACCGCACCAAGACGAACTGCAGCAAAGGCATGTTCAAGGATTTCTCCTGAATCAAGAAACCGAAGTGTGGGAATCGAGGTGATTGGAGCAGTTGTAGTCCACACTGCAAACGATGACACCCAA | *Leishmania spp.* | 8.00E-44 | 87.82% |
| R21-023 | *Tadarida brasiliensis* | GTACTGGGGCGTCAGAGGTGAAATGTTTCAACCGCACCAAGACGAACTGCAGCAAAGGCATGTTTAGGGTGCTCCTGAATCAAGAAACCGAAGTGTGGGGATCGAAGATGATTGGAGCCGTTGTAGTCCACACTGCAAACGATGACACCCAA | *Leishmania spp.* | 1.00E-41 | 87.18% |
| R21-068* | *Tadarida brasiliensis* | GTACTGGGGCGTCAGAGGTGAAATTTTTAGACCGCACCAAGACGAACTACAGCGAAGGCATTCTTCAAGGATACCTTCCTCAATCAAGAACCAAAGTGTGGGGATCGAAGATGATTAGAGACCATTGTAGTCCACACTGCAAACGATGACACCCAA | *Leishmania spp.* | 1.00E-72 | 98.71% |
| R21-078* | *Tadarida brasiliensis* | GTACTGGGGCGTCAGAGGTGAATTTTTAGACCGCACCAAGACGAACTACAGCGAAGGCATGTTCAAGGACTTCCTGAATCAAGAACCAAAGTGTGGGGATCGAAGATGATTAGAGACCATTGTAGTCCACACTGCAAACGATGACACCCAA | *Leishmania spp.* | 2.00E-59 | 94.19% |
| R21-014 | *Tadarida brasiliensis* | - | *Leishmania spp.* | N/A | N/A |
| R21-042* | *Tadarida brasiliensis* | - | *Leishmania spp.* | N/A | N/A |

*Specimens with co-infections of *T. cruzi* and *Leishmania spp.*
